# The role of metabolic reprogramming in oocyte development: research progress and prospects

**DOI:** 10.3389/frph.2026.1803167

**Published:** 2026-05-21

**Authors:** Yawen Li, Yu Liu, Jing Jin

**Affiliations:** 1 School of Medicine, Wuhan University of Science and Technology, Wuhan, China; 2 Department of Gynecology, Hubei Maternal and Child Health Hospital, Wuhan, China

**Keywords:** assisted reproductive technology, early-onset ovarian insufficiency, *in vitro* maturation, metabolic reprogramming, mitochondrial function, oocyte growth and development, pentose phosphate pathway, polycystic ovary syndrome

## Abstract

**Introduction:**

In oocyte growth and development, metabolic reprogramming serves as a core process by which cells adjust their metabolic patterns—including glucose metabolism, lipid metabolism, and amino acid metabolism—to adapt to developmental cues, differentiation, and environmental stress. This reprogramming is essential for maintaining energy homeostasis and supporting biosynthesis. A synergistic and precisely regulated metabolic network provides the necessary energy, reducing equivalents, and biosynthetic precursors for oocyte maturation, fertilization, and early embryonic development.

**Methods:**

This paper systematically reviews the key roles and regulatory mechanisms of the three major metabolic pathways in oocyte development. A literature–based synthesis was conducted, integrating findings from studies on oocyte metabolism and metabolic disorders.

**Results:**

The results show that metabolic dysregulation—associated with diseases such as diabetes, obesity, polycystic ovary syndrome, and early-onset ovarian insufficiency—impairs oocyte quality and embryonic development potential by disrupting ovarian microenvironmental homeostasis.

**Discussion:**

Furthermore, this review discusses potential strategies to increase the success rate of assisted reproduction, including targeted metabolic intervention, multi–omics integration analysis, and mitochondrial function optimization, thereby providing a theoretical basis for clinical practice and scientific research in reproductive medicine.

## 1 Introduction

The egg, as the most distinctive cell in the female reproductive system, relies on highly coordinated cellular metabolism and microenvironment support for its growth and development. Metabolic reprogramming, as a core mechanism for cells to adapt to changes in the internal and external environment, plays a key role in oocyte maturation, energy supply, REDOX balance, and epigenetic regulation. In recent years, with the deepening of the intersection of reproductive medicine and metabolic research, the molecular mechanisms of oocyte metabolic regulation have gradually been revealed, especially in patients with advanced age, metabolic diseases and reproductive endocrine disorders, metabolic disorders have become important factors affecting oocyte quality and fertility outcomes. This paper aims to systematically summarize the mechanism of metabolic reprogramming in oocyte growth and development, sort out the synergistic network of glucose, lipid and amino acid metabolism, analyze the association between metabolic abnormalities and reproductive diseases, and look forward to potential strategies for improving oocyte quality and assisted reproductive efficiency through metabolic regulation, with the aim of providing references for clinical practice and research.

## 2 Metabolic reprogramming

Metabolic reprogramming refers to the process by which cells adjust their metabolic patterns during development, differentiation, or under environmental stress to maintain energy homeostasis and biosynthesis (1). The core pathways include glucose metabolism, lipid metabolism, and amino acid metabolism. Metabolic reprogramming is the central coordinator of oocyte growth and development, ensuring that oocytes receive energy and raw materials at the right time and in the right way, while protecting their genetic and epigenetic integrity and reserving the necessary “maternal metabolic capital” for subsequent fertilization and embryonic development.

## 3 Core pathways of metabolic reprogramming

### 3.1 Glucose metabolism

Glucose metabolism is the most core energy metabolism and substance synthesis pathway in living organisms, involved in three metabolic pathways: glycolysis, pentose phosphate, and oxidative phosphorylation.

#### 3.1.1 Glycolysis: the fundamental pathway for cellular energy supply and its multiple regulatory functions

Glycolysis is the main metabolic pathway of highly conserved proliferating cells (2), in which glucose is phosphorylated and cleaved into two three-carbon molecules (glyceraldehyde 3-phosphate) under anaerobic conditions through the catalysis of hexokinase to consume Adenosine triphosphate(ATP), generating ATP and Nicotinamide adenine dinucleotide (NADH), and ultimately forming pyruvate (3, 4). Glycolysis is a major source of ATP production in various cellular states (5), such as hypoxia, and is particularly important for maintaining bioenergy homeostasis in the most abundant cell types in the human body. For example, ATP levels directly determine the activity of AMPK. AMPK is the cell's energy “sensor,” and ATP prevents AMP/ADP from binding to AMPK, thereby inhibiting its activation (6–9). The glycolytic product NADH and its oxidized form NAD+ play a crucial role in maintaining REDOX balance (NAD+/NADH) by promoting REDOX reactions to ensure the effective functioning of key metabolic pathways such as glycolysis and oxidative phosphorylation, for example, NAD+ deficiency affects the occurrence of oxidative stress in various diseases, While raising NAD+ enhances antioxidant capacity by increasing glutathione (GSH) levels and antioxidant enzyme activity, it has a protective effect (10). NAD+/NADH also plays a key role in a variety of cellular processes, many of which are mediated by enzymes that consume NAD+. For example, NAD+ is a necessary substrate for the Sirtuin family of deacetylases (11), which are involved in a variety of biological processes, including regulating metabolism and inflammation (12), and promoting cell lifespan by deacetylating lysine residues on histones and other proteins, thereby altering gene expression (13–15). In addition to its role in promoting Sirtuin deacetylation, NAD+ also regulates other DNA repair pathways by activating NAD+ consuming enzymes, PARPs, and sirtuins (16, 17). Therefore, cellular NAD+ depletion and a decrease in the NAD+/NADH ratio are associated with cellular senescence, aging, and the onset of age-related diseases (17). Another glycolytic product, pyruvate, enters the mitochondria under aerobic conditions and is oxidized to acetyl-coA, which binds to oxaloacetic acid to initiate the tricarboxylic acid (TCA) cycle, as well as Oxidative phosphorylation(OXPHOS) that can produce 36 ATP, while under anaerobic conditions, pyruvate is reduced to lactic acid by lactate dehydrogenase A (LDH-A) in the cytoplasm. Lactic acid is then excreted to the extracellular space via monocarboxylic acid transporters (MCTs) (18). In summary, glycolysis is not only the fundamental pathway for providing energy to cells, but also plays a significant role in cell adaptation to the environment, maintaining homeostasis and aging through its metabolites, which are widely involved in energy sensing, REDOX regulation and epigenetic modification.

#### 3.1.2 Pentose phosphate pathway (PPP): the core pathway for substance synthesis and redox homeostasis

The pentose phosphate pathway (PPP) is the processby which glucose-6-phosphate is metabolized by glucose-6-phosphate dehydrogenase (G6PDH) to produce nicotinamide adenine dinucleotide phosphate (NADPH) and phosphate nucleotide (PRPP) (19–22) and it is a fundamental component of cellular metabolism. The primary biochemical function of PPP is the biosynthesis of nucleic acid and amino acid sugar phosphate precursors to sustain cell survival. PPP, for example, maintains a core role in central metabolism that provides RNA skeleton precursors ribose 5-phosphate and red sugar 4-phosphate as aromatic amino acid precursors, where ribose 5-phosphate is essential for the formation of RNA and DNA skeletons, and red sugar 4-phosphate is a precursor for histidine biosynthesis. It is used for various aromatic metabolites in the protozoa of aromatic amino acids and plays a role in vitamin B6 metabolism (23–25). The function of PPP is closely related to providing the biochemical reduction equivalent NADPH, which maintains cytoplasmic stability and REDOX homeostasis by reducing glutathione (GSH), making PPP play an important role in maintaining REDOX homeostasis. It plays a crucial role in providing the required reducing power for a variety of biosynthetic reactions, detoxification processes, and cellular antioxidant defenses (26, 27). It is necessary to briefly explain here that reactive oxygen species (ROS), as active molecules produced during cellular metabolism, have a dual role in cellular processes: under physiological conditions, they can act as signaling molecules involved in cell signal transduction, oocyte maturation, and other normal physiological processes; meanwhile, a moderate accumulation of ROS can also participate in cellular stress responses and regulate cell metabolism to adapt to the external environment. This is the core significance of ROS production in cells. For example: A newly discovered enzyme—yeast NADPH oxidase YNO1, similar to mammalian NADPH oxidase, oxidizes NADPH to superoxide, which increases superoxide levels tenfold when YNO1 is overexpressed in wild-type cells. However, after the absence of ZWF1, the superoxide level no longer increased, suggesting that PPP compensates for the increase in NADPH consumption caused by YNO1 overexpression (28). PPP is highly flexible and dynamic, able to adapt to different nutrient supplies and stress conditions, which coordinates these functions and must meet the demands of cellular metabolism in a constantly changing environment. PPP activity increases rapidly when cells are exposed to oxidants, for example. In the first few seconds of the oxidative burst, glycolytic enzymes, glyceraldehyde 3-phosphate dehydrogenase (GAPDH) (29), and pyruvate kinase (PK) (30) are inactivated, causing glycolysis to be blocked, while the flow of PPP continues (31). Subsequently, cells maintain the enhanced state of PPP through transcriptional regulation and enzyme activity modifications such as boosting G6PDH activity (32). This regulatory mechanism has a dual meaning: under normal circumstances it prevents excessive generation of NADPH and metabolic intermediates and reduces carbon loss; Under stress conditions, it guarantees the cell's abilityto respond quickly (29, 31). PRPP serves as a substrate for the new synthesis and resynthesis of purine nucleotides. Its contribution to nucleotide formation is crucial for cellular function, particularly in supporting nuclear maturation (33, 34). PRPP, for example, is an essential substrate in the salvage synthesis reaction catalyzed by purine phosphoribosyltransferase. These enzymes use PRPP to phosphoribosylate adenine or 6-oxpurine (such as hypoxanthine, xanthine, guanine) to generate the corresponding nucleotides (AMP, IMP, XMP, GMP), thereby recovering the free purine base (34, 35).

#### 3.1.3 Oxidative phosphorylation (OXPHOS): the core terminal pathway of cellular energy metabolism

Oxidative phosphorylation (OXPHOS) is an enzymatic process linked by an electron transfer coupled to ATP synthesis via an electrochemical transmembrane gradient, which takes place in mitochondria and is the final biochemical pathway for ATP production. Most cells produce the majority of adenosine triphosphate (ATP) through OXPHOS, a pathway that utilizes the energy released from the oxidation of nutrients (18). Mitochondrial OXPHOS require the coordinated action of the electron transport chain (ETC) complex located on the inner mitochondrial membrane and ATP synthase (36). The net effect of electron transport is to establish and maintain a strong proton gradient to power ATP synthesis, and ATP synthase (complex V) is the terminal execu tor of oxidative phosphorylation and plays a crucial role in energy production (37, 38). It is the main ATP-generating process, which requires the participation of oxygen (39). OXPHOS can directly drive the continuous catabolic processes such as the tricarboxylic acid cycle and fatty acid β-oxidation by consuming NADH and FADH_2_, while the cellular energy state (ATP/ADP ratio) serves as a core signal to reverse-regulate upstream pathways such as glycolysis and insulin secretion, achieving a dynamic balance of the metabolic network throughout the cellof the metabolic network throughout the cell (40). The electron donors produced by metabolic pathways such as glycolysis, the TCA cycle and β-oxidation provide fuel for ETC. Similarly, ETC activity affects a variety of processes beyond energy balance, such as the electron transport chain being one of the main sources of reactive oxygen species (ROS) within the cell, which act as important signaling molecules involved in regulating hypoxia adaptation, immune response, and autophagy (41). In summary, oxidative phosphorylation is at the core of cellular energy metabolism. It efficiently synthesizes ATP in mitochondria, and its electron transport chain is not only an energy engine but also regulates overall cellular metabolism and stress by generating signaling molecules such as ROS.

In summary, glucose metabolism provides cells with ATP, biosynthetic precursors, reducing power (NADPH), and participates in signal transduction and REDOX balance.

### 3.2 Lipid metabolism

Lipid metabolism refers to the complex process in which fats in organisms are digested, absorbed, synthesized and decomposed by various enzymes, which can maintain normal physiological functions of the human body. The metabolites include lipoproteins, fatty acids and cholesterol (42, 43). Lipoproteins, secreted by the liver and small intestine, have the primary function of transporting lipids [cholesterol, triglycerides (TGs) and phospholipids (PLs)] to peripheral tissues and returning lipids and cholesterol to the liver for clearance or recycling (44). Fatty acids are the simplest type of lipids and are part of many complex lipids. Fatty acids, for example, are used to synthesize energy-rich triglycerides and signaling molecules such as prostaglandins (45). In addition, fatty acids are broken down into acetyl-coA through β-oxidation, providing fuel for energy generation in the tricarboxylic acid cycle. The ATP energy stored in fatty acids is almost 3.5 times that of glucose and twice that of protein, making lipids an efficient source of energy (46). In addition to being involved in energy storage and cellular signaling, fatty acids are also associated with intellectual development, growth and health, memory and physiological functions (47). Cholesterol is the basic skeleton that makes up all cell membranes and organelle membranes (48), and its composition and fluidity directly affect the function of membrane proteins and the formation of immune synapses. Cholesterol can exist in the blood in the form of very low-density lipoprotein (VLDL), low-density lipoprotein(LDL), and high-density lipoprotein (HDL). Cholesterol is involved in the formation of bile acids and cell membranes, as well as in the synthesis of steroid hormones such as cortisol, aldosterone, testosterone, estradiol and progesterone, 1, 25-dihydroxyvitamin D3 (49). In addition, there are dietary lipids, mainly triglycerides, which account for about a third of a person's total calorie intake (50). After being digested by enzymes, the lipids are absorbed by small cells in the small intestine. A small amount is used for energy production or membrane lipid formation, while most of the absorbed lipids are packaged in cytoplasmic lipid droplets for short-term storage within the cell, or assembled into chylomicrons for transport throughout the body (51). In summary, lipid metabolism plays a central role in sustaining life activities, involving not only the efficient supply and storage of energy, but also closely involved in cell construction, signal transduction, and the regulation of various physiological functions.

### 3.3 Amino acid metabolism

An amino acid is an organic molecule that contains an amino group and a carboxyl group and is attached to a carbon atom. Each amino acid also has a unique side chain that gives different properties and functions (52). Amino acids are important nutrients, and amino acid metabolism is used in many processes of cell proliferation, growth, and cell function. Amino acid metabolism supports cells' adaptability in rapid proliferation or stress states by providing biosynthetic precursors, maintaining REDOX balance, acting as cellular signaling, and regulating epigenetics. Amino acids and their metabolites are direct raw materials for the synthesis of nucleic acids (purines, pyrimidines), proteins, lipids, and other biomolecules such as glutathione (53). For example, certain amino acids (such as glycine and serine) are important metabolic pre cursors of the “nitrogen-containing bases” in the synthesis of nucleotides (nucleic acid monomers) (54). Mammals use 20 kinds of amino acids for protein synthesis. Glutamine replenishes carbon atoms for the tricarboxylic acid cycle (TCA cycle), supporting lipid synthesis (55). Glutamic acid provides the γ-carboxyl group to form a unique γ-peptide bond with the amino group of cysteine, which is key to the stability of glutathione (GSH) (56). Amino acids not only provide carbon and nitrogen sources for the synthesis of biomolecules (57), but their metabolites also enter the tricarboxylic acid cycle (TCA cycle) to supply adenosine triphosphate (ATP) to cells (58). Under specific conditions (such as glucose deficiency), amino acids (such as glutamine, branched-chain amino acids) can supply ATP to the cell either by converting to TCA cycle intermediates (known as “complementing”) or by oxidative phosphorylation. Amino acid metabolism regulates REDOX balance (55). Glutamine is an oxidizable substrate that maintains TCA cycle activity and cell survival (59). Among them, the glutamine-glutamine-glutathione axis is one of the most important antioxidant systems within the cell, and an enhanced metabolic flow helps clear reactive oxygen species (ROS) and protect cells from oxidative stress damage (55). Some amino acids themselves are signaling molecules. Leucine acts as a signaling molecule to directly activate the mTORC1 pathway, promoting protein synthesis and cell growth (60). Arginine and nitric oxide (NO) signaling are involved in regulating angiogenesis and immune function (61, 62). Intermediate metabolites produced by amino acid metabolism are also substrates or cofactors of epigenetic modified enzymes [for example, α -ketoglutaric acid (α-KG) is a necessary cofactor for TET and JMJ family demethylases], and changes in their levels directly affect the methylation status of histones and DNA, thereby regulating gene expression (63). In conclusion, amino acids are far more than just raw materials for the synthesis of proteins and the like. Their metabolic networks are deeply involved in and support cell proliferation, adaptation, and functional execution by providing precursors for the synthesis of life macromolecules, maintaining REDOX balance, acting as signaling molecules, and regulating epigenetics.

## 4 The role of metabolic reprogramming in oocyte development

### 4.1 The role of glucose metabolism in egg development

A large amount of ATP is needed during oocyte maturation, and glucose metabolism is crucial for oocyte development ability, mainly dependent on glycolysis and OXPHOS production (64). Kirillova et al. suggested that oocyte quality is directly related to ATP availability (65). Glycolysis is the main energy source for oocyte meiosis. Oocytes are highly dependent on the metabolic support of granulosa cells (GCs) due to the lack of key glycolytic enzymes (66). Almost all the glucose consumed by oocytes is metabolized in granulosa cells through glycolysis to produce ATP and the glycolytic product pyruvate (67, 68). Glycolytic activity in the GCs peaks 8 h after the LH peak, in sync with oocyte maturation. After the LH peak is triggered, mitochondrial respiration and glycolysis in GCs are significantly upregulated to provide energy support for oocytes, and these metabolic changes directly affect oocyte maturation and subsequent embryonic development (69). Low glycolytic activity leads to a decline in oocyte development ability (70), and inhibition of glycolytic can result in maturation disorders (71) and abnormal oocyte defects and epigenetic modifications (72). Studies have found that a high sugar environment may lead to oocyte arrest or a decline in quality (73). Instead, increased glycolytic activity leads to increased blastocyst development (74, 75). For example, an increase in glycolysis typically observed at maturity is delayed in oocytes of adolescent sheep and cows with lower developmental capacity (76, 77). In addition, advanced age oocytes (GV and MI stages) exhibit enhanced glycolytic metabolism (elevated levels of glucose, pyruvate and lactate), while relying on the adenosine rescue pathway (reduced phosphocreatine and AMP) and ATP provided by surrounding granulosa cells as an alternative energy source to promote oocyte development (78). Additionally, pyruvate, a glycolytic product, plays a key role in oocyte growth and development. There is a division of labor in pyruvate metabolism between oocytes and the surrounding granulosa cells. Granulosa cells inhibit further oxidation of pyruvate by highly expressing PDK enzyme, allowing it to accumulate; Oocytes, on the other hand, use the active PDH enzyme to efficiently convert pyruvate into acetyl-coA, which enters the tricarboxylic acid cycle to generate A large amount of ATP to support their own maturation (79, 80). Therefore, glycolysis not only provides the core driving force for oocyte maturation, but the effective conversion of its metabolite pyruvate is also crucial for ensuring the ability of the embryo to develop later.

In fact, during oocyte growth and development, glycolysis and OXPHOS can interconvert in some cases, and the ATP generated by glycolysis and the glycolytic product pyruvate transport these energy substrates to the oocyte through gap junctions to support OXPHOS. Among them, gap junctions are primarily composed of the connexin proteins Cx37 and Cx43. Both serve as key physical connections between granulosa cells and oocytes and act as important channels for the bidirectional transfer of nutrients and signaling molecules. They are crucial for energy supply and metabolic regulation during oocyte maturation. Cx37 mediates direct communication between oocytes and granulosa cells and can also form communication channels among granulosa cells adjacent to the oocyte, ensuring efficient transport of energy substrates. Cx43, on the other hand, plays an important role in folliculogenesis and the development of the embryonic reproductive ridge. Both work together to maintain the normal function of gap junctions, providing the structural basis for the transport of glycolytic products to oocytes. During the growth of oocytes, they also exhibit metabolic characteristics similar to the Warburg effect, namely enhanced glycolysis and reduced OXPHOS capacity. The study found that when melatonin synthesis was inhibited (such as by siAANAT knockdown), oocytes shifted from OXPHOS to glycolysis (Warburg effect), as indicated by upregulated expression of key glycolytic genes (PGD, LDHA), but ATP production decreased. Supplementing melatonin reversed this metabolic transition. Restore the energy metabolism pattern dominated by OXPHOS (81). Research has also shown that during oocyte maturation, fertilization, and embryonic development, the demand for ATP continuously increases, which also drives oocytes to shift from glycolysis to oxidative phosphorylation (OXPHOS) as the main energy source for maintaining normal physiological functions (79, 82).

Oocytes are highly dependent on oxidative phosphorylation for ATP production, and mitochondria are crucial in this process, as oocytes are the cells richest in mitochondrial content. During oocyte maturation, mitochondria generate large amounts of ATP (adenosine triphosphate) through oxidative phosphorylation (OXPHOS), serving as a key source of energy supply. Ensuring adequate mitochondrial function is therefore essential (76). Reactive oxygen species(ROS), as a by-product of OXPHOS, is also an energy source for oocytes (83). Drug treatments and mitochondrial supplementation, for example, can be used to improve oocyte quality and enhance fertility by increasing ATP production and reducing ROS levels (84, 85). Although a small amount of ROS is useful and can act as a signal within cells, high concentrations of reactive oxygen species can cause damage. ROS can also react with a variety of biomolecules such as lipids and nucleic acids, causing cell damage and oxidative stress, damaging oocytes and reducing their quality (86). The accumulation of advanced glycation end products (AGEs) can trigger oxidative stress and mitochondrial dysfunction, further accelerating oocyte aging (87), while mitochondrial functional defects (such as reduced membrane potential, insufficient ATP) can lead to oocyte maturation failure (81), and mitochondrial DNA mutations or functional abnormalities can reduce oocyte developmental potential (69). There is also evidence that a decrease in ATP levels can lead to meiosis errors, which in turn alter the number of chromosomes in oocytes and cause genetic diseases disorders (88). In addition, Rodriguez Nuevo et al. suggested that human oocytes maintain ROS-free metabolism by eliminating complex I and reshaping the mitochondrial electron transport chain (89). In patients with reproductive disorders, quantitative defects (such as exhaustion) and qualitative defects (such as mutations) were found in mtDNA (90), suggesting that mitochondrial deficiency may lead to the failure of oocyte maturation (91). Pasquariello et al. found that in older human oocytes, mitochondrial activity was reduced, mitochondrial membrane potential was lowered, and the number of mitochondrial DNA copies was increased compared with young oocytes (92). Maternal aging is associated with mitochondrial dysfunction, which impures oocyte quality and increases the risk of chromosomal abnormalities in embryos (93, 94). In summary, the efficient progress of oxidative phosphorylation is highly dependent on the structural and functional integrity of mitochondria, both of which together affect oocyte energy metabolism and profoundly influence its developmental outcomes.

The key role of PPP in the delicate balance of cellular processes required for oocyte development and maturation in culture. PPP is crucial for ensuring oocyte quality and embryonic developmental potential. The pathway not only provides the crucial NADPH to combat oxidative stress and maintain the cell's reduced state, but also participates in nucleotide synthesis, thereby directly supporting oocyte nuclear maturation and cytoplasmic maturation (95–98). The study found that granulosa cells around oocytes metabolize glucose through PPP and produce substances such as NADPH that protect against oocyte senescence. Even when glycolysis is inhibited, PPP can still function independently; Conversely, if PPP is inhibited, glycolysis will also cease completely. Supplementation of PPP intermediates (if sugar-6-phosphate) can effectively alleviate oocyte sen escence caused by PPP inhibition (99–101). In addition, the activity of PPP is regulated by factors such as PPARδ, and its activation can enhance glucose metabolism through PPP, helping to improve the adverse effects of metabolic abnormalities such as hyperglycemia on reproduction (102). Clinical studies also suggest that the estrogen receptor G6PDH and the PPP axis can simultaneously reduce glycolytic metabolites and PPP intermediates, leading to metabolic imbalance in endometrial tissue and resulting in infertility (103). In addition, the energy (ATP) and reduction equivalent (NADPH) produced by PPP support meiosis, and its abnormalities can lead to oocyte defects and epigenetic modification abnormalities (72). Therefore, the pentose phosphate pathway (PPP) plays an irreplaceable role in oocyte maturation and embryonic development by providing energy, reducing power and synthetic raw materials, and its functional imbalance is directly associated with reproductive disorders.

In summary, glycolysis, oxidative phosphorylation, and the pentose phosphate pathway work together to form the energy and material basis of oocyte maturation and embryonic development, and their fine balance and timely conversion are key to reproductive success.

### 4.2 The role of lipid metabolism in oocyte development

Oocyte quality and developmental ability are closely related to lipid metabolism, which provides a powerful source of energy and is extremely important during oocyte maturation. Fatty acids produce 3.5 times more energy than glucose, so this metabolic process that generates ATP is highly efficient (104), especially the β-oxidation of fatty acids (105, 106). Fatty acid β-oxidation is a key process in ATP production, especially in early embryos where glycolytic activity is low and dependent on fatty acid oxidation for energy supply (107). Oocytes convert fatty acids to acetyl-coA through β-oxidation and enter the tricarboxylic acid cycle (TCA) to produce ATP (66). Fatty acids have the highest ATP content in the fatty acid β-oxidation (FAO) pathway compared to other macronutrient categories, and an increase in β-oxidation raises NADH levels, providing the energy supply needed for oocyte maturation (108, 109). The study found that β-oxidation is also necessary for the recovery of meiotic division and nuclear maturation of oocytes in mice, cattle and pigs (110, 111). And an increasing number of reports suggest that inhibiting fatty acid oxidation would undermine the recovery and developmental potential of oocyte meiosis (108, 112). In addition, in a variety of species, the inhibition of β-oxidation during oocyte maturation indicates that this metabolic pattern plays a significant role in subsequent embryonic development. For example, immature bovine oocytes exposed to methyl palmoxirate [which inhibits CPT1 (carnitine palmitoyltransferase-1)] showed reduced oxygen consumption and impaired ability to develop to the blastocyst stage (105). Similarly, treatment of porcine oocytes during maturation with methyl palmitoyl isopropyl oxime or mercaptoacetic acid (an inhibitor of 3-hydroxy-CoA dehydrogenase in the β-oxidative spiral) leads to blocked fertilization and decreased blastocyst development (106). Studies have shown similar sensitivity of mouse oocytes to β-oxidation inhibition, in which treatment of COCs with the Cpt1 inhibitor etomoxir significantly reduced β-oxidation levels and significantly decreased the number of blastocyst embryos after fertilization (108). In addition to β-oxidation, free fatty acids and their derivatives are also released as signaling molecules under strict developmental controls, participating in a variety of cellular processes by regulating gene transcription, protein modification, and enzyme activity (113). For example, prostaglandins/leukotrienes synthesized by fatty acid catabolism may be able to promote autocrine in oocyte meiosis (114). There are also studies showing significant differences in the fatty acid composition of human oocytes compared to adipose tissue in other parts of the body—oocytes have a higher content of saturated fatty acids (about 27%). The same is true in cattle: saturated fatty acids are also abundant in their oocytes or cumulus-oocyte complexes (COCs) compared to granulosa cells and blood from the same animal. This suggests that oocytes may have the ability to selectively absorb fatty acids and may even synthesize fatty acids on their own to meet their specific energy storage and metabolic needs (115–117). Therefore, fatty acids are not only an efficient source of energy for oocytes, but also key regulators of their maturation and embryonic development, with their unique composition and metabolic patterns jointly ensuring the smooth progress of the reproductive process.

Oocytes are renowned for their lipid reserves, which accumulate in droplets. During the production of mammalian eggs, lipid droplets are stored in the cytoplasm, although the lipid droplet content varies greatly among different species (118). Lipid droplets provide a crucial energy reserve for periods of nutrient scarcity and contribute to energy production and cellular signaling (119, 120). During oocyte maturation and early embryonic development, lipid droplets act as an important raw material to generate high energy to support cell division and growth (105, 121). Lipid accumulation in an *in vitro* maturation (IVM) environment, for example, can lead to oxidative stress and apoptosis, thereby affecting the developmental potential of the oocyte (64). Studies have shown that PCB2 improves the developmental capacity of oocytes by activating the PPARγ/UCP1 pathway, promoting mitochondrial uncoupling and lipid catabolism, and reducing lipid accumulation in oocytes (64). During the maturation of oocytes, specific changes occur in the distribution of lipid droplets within the cells. The study found that in porcine oocytes, lipid droplets that mature *in vitro* tend to be distributed at the cell margins (106). In bovine oocytes, the number of lipid droplets increases slightly but significantly after *in vitro* maturation (122). In mouse oocytes, whether mature *in vivo* or *in vitro*, lipid droplets undergo structural reorganization and tend to cluster towards the central region of the cell (123–125). The increase in the number of lipid droplets during oocyte maturation is crucial for energy reserves. However, the relationship between lipid droplet content and oocyte developmental potential is complex and not simply “the more the better” or “the less the better”, but excessive accumulation may lead to oxidative stress and apoptosis (126). In some mouse studies, it was found that oocytes with poorer developmental ability sometimes contained more lipid droplets, but they were often accompanied by other cytoplasmic defects as well (127). Therefore, lipid droplets, as an energy reserve of oocytes, are crucial in terms of their abundance and distribution for maturation and embryonic development, but proper regulation is far more critical than simply increasing or decreasing their numbers.

Cholesterol exists in the form of lipoproteins, and lipoprotein particles (such as HDL, LDLs, and VLDLs) can carry fatty acids stored in the form of triglyceride molecules or triglycerides into the bloodstream, but LDL and VLDL present in follicular fluid are likely derived from local synthesis in the ovaries. Analysis of existing studies suggests that high-density lipoprotein (HDL) is dominant in mammalian follicular fluid, while low-density lipoprotein (LDL) or very low-density lipoprotein (VLDL) is rarely or even undetectable (128, 129). However, some studies have detected LDL or VLDL in the follicular fluid of some women. This suggests that the lipoproteins may not come from the blood but are synthesized and secreted by granulosa cells or luteal cells within the ovaries themselves (130, 131). Therefore, high-density lipoprotein (HDL) granules play a significant role in steroidogenesis in granulosa cells and follicular membrane cells and are a major source of cholesterol (132). Animal experiments and some human observations suggest that HDL and its main apolipoprotein ApoAI are crucial for maintaining fertility and improving embryo quality, possibly by providing cholesterol feedstock or providing antioxidant protection (130, 133, 134). Other clinical studies have reached the opposite conclusion, finding that high levels of HDL and ApoAI in follicular fluid may actually reduce oocyte maturation and embryo quality (135, 136). Therefore, it remains uncertain whether it is the lipid substrate carried by the lipoprotein itself, such as triglycerides, that plays a role, or whether the apolipoprotein component affects oocyte quality through mechanisms such as antioxidation.

All in all, lipid metabolism is closely linked to the quality of oocyte development. Fatty acids and their β-oxidation are not only efficient energy sources, but also directly regulate oocyte maturation and early embryonic development; The timely use and dynamic distribution of lipid droplets jointly affect the developmental potential of oocytes; The source, composition and function of lipoproteins in the follicular fluid still require further clarification of their balancing role between energy supply and cell protection.

### 4.3 The role of amino acid metabolism in oocyte development

Early studies have shown that oocytes, being the largest cells in the body, depend on efficient nutrient support for their maturation and quality, with the active transport of amino acids playing a crucial role in nourishing oocytes. Oocytes can regulate the metabolism of surrounding granulosa cells through paracrine factors (such as GDF9 and BMP15), particularly by modulating the uptake and metabolism of amino acids and glucose, thereby providing nutritional support to the oocytes (137, 138). Among them, glutamine is considered an efficient energy substrate that supports oocyte development. For example: Glutamine can act as the sole energy source to initiate meiotic recovery in mouse oocytes within the COC, although not through the MII stage (139), adding glutamine to the medium promotes oocyte maturation in cattle, hamsters, dogs, rabbits, and rhesus monkeys (140–143). In addition, amino acids (such as glutamine) enter the TCA cycle by generating α-ketoglutaric acid, which is an important fuel for mitochondrial ATP production and directly affects the developmental potential of oocytes (65). The addition of amino acids such as glutamine also enhances antioxidant capacity, and GLN regulates ROS as an important source of glutathione (GSH) synthesis to improve oocyte quality (144, 145). Glycine plays a positive role in promoting *in vitro* maturation and development of porcine oocytes. Sequencing results indicated that the treatment significantly regulated the expression of cell membrane and mitochondrial-related genes, suggesting that glycine may function by maintaining the integrity of the biological membrane structure. Further studies revealed that glycine could reduce reactive oxygen species (ROS) -induced lipid peroxidation, thereby contributing to the improvement of cell membrane structure and function (146). In addition, glycine can also regulate the volume of oocytes. The glycine transporter GLYT1 is quiescent in immature GV oocytes, which also contain very little endogenous glycine. However, GlyT1-mediated glycine transport is activated in oocytes within a few hours after ovulation begins, and oocytes can use glycine to regulate volume at the same time (147). In addition, multiple studies have shown that the lack of SHMT1 in the SGOC pathway only leads to no obvious phenotype in mouse oocytes, while the lack of SHMT2 leads to direct death of oocytes, suggesting that SHMT2 in mitochondria may be more important, thereby compensating for the loss of cytoplasmic SHMT1 (148–150). In summary, amino acids not only provide fuel and protection for oocyte maturation directly as energy substrates and antioxidants, but also jointly shape the sophisticated microenvironment that undergoes oocyte development by regulating key processes such as metabolism, membrane structure, and cell volume.

## 5 Metabolic reprogramming abnormalities and reproductive diseases

The maturation and quality of eggs are highly dependent on the precise metabolism of three types of nutrients: glucose, lipids, and amino acids. The homeostasis of the ovarian microenvironment is disrupted when the body is in metabolic disorder due to diabetes, obesity, or reproductive endocrine disorders such as PCOS and POI. This directly impairs the function of granulosa cells, disrupts the energy supply and antioxidant defense of oocytes, and ultimately leads to a decline in their developmental potential, impaired embryo quality, and deteriorating reproductive outcomes.

Abnormal glucose metabolism can affect the growth and development of oocytes. Diabetes is typically characterized by elevated blood sugar levels. Studies have found that in a variety of ovarian cells (primal/primary follicular granulosa cells, secondary/lumen follicular granulosa cells, luteal cells) in diabetic mice, basal metabolic pathways such as fatty acid metabolism, n-glycan synthesis, pyruvate metabolism, and glycolysis are inhibited to varying degrees (151). It has also been found to cause changes in mitochondrial structure and space in oocytes, which further leads to differences in ATP content (152), thereby affecting oocyte quality, embryonic development and implantation (153, 154). Insulin resistance, which is characterized by reduced insulin sensitivity and impaired glucose uptake and utilization in cells, disrupts glucose metabolism and negatively affects oocyte maturation and embryo development. Likewise, insulin deficiency reduces glucose supply to germ cells, hinders energy production and lowers fertility. Studies have found that insulin-related metabolic disorders are associated with both polycystic ovary syndrome (PCOS) and early-onset ovarian insufficiency (POI) (155). PCOS is characterized by disdisorder of follicular development, including small anterior follicular accumulation, and patients often show disorders of glycolysis and pyruvate metabolism in follicular fluid, with an increased risk of diabetes (156), hyperandrogenism and insulin resistance. It may lead to a reduced ability of granulosa cells to take up and utilize glucose, along with decreased expression of glycolycle-related genes, which together impair the normal maturation of oocytes (157–159). PCOS patients cause metabolic defects in ovarian granulosa cells, leading to reduced expression of hypoxia-inducible factor HIF-1*α*, weakening granulosa cells' glycolytic capacity, reducing energy substrates for oocytes, and also causing a deficiency of the mitochondrial key protein SIRT3, Increased reactive oxygen species, decreased membrane potential, and reduced activity of G6PDH, a key enzyme in the pentose phosphate pathway, all of which disrupt the normal insulin signaling pathway and lead to reduced fertilization ability of oocytes in POC patients, resulting in clinical outcomes of *in vitro* fertilization and embryo transfer (IVF-ET) (157, 160). A typical feature of POI patients is premature ovarian failure (161). The study found that it was accompanied by significant metabolic abnormalities such as elevated blood sugar and insufficient insulin secretion (158, 159). The latest research suggests that enhancing granulosa cell proliferation and glycolysis by activating the HIF1α/CX43 pathway could be a potential direction for supporting follicular development and improving ovarian function. Obese patients may reduce the expression of glucose metabolism markers, including PPARγ -mediated insulin-degrading enzymes, retinol-binding protein 4 and GLUT4, ultimately damaging ovarian quality (162, 163). In addition, clinical data show that obese women have lower live birth rates after receiving assisted reproductive technology than women of normal weight (164). Studies have also found that older females exhibit an aging phenotype in mature oocytes with increased oxidative stress and mitochondrial dysfunction, and as women age, the efficiency of DNA damage repair decreases, resulting in an increase in DNA mutations and the frequency of DNA damage, which in turn leads to a higher incidence of chromosomal integrity loss and misalignment (165). The accumulation of mitochondrial DNA mutations over time leads to mitochondrial defects, followed by the accumulation of intracellular ROS, which in turn causes more DNA damage, creating a vicious cycle that eventually leads to apoptosis (166).

Abnormal lipid metabolism can also affect oocytes, which are closely associated with a decline in oocyte quality. For example, the content of free fatty acids in the eggs of older women is significantly reduced, while the accumulation of lipid droplets increases, leading to impaired mitochondrial function and elevated levels of oxidative stress. Abnormal lipid metabolism also affects the synthesis of prostaglandin (PGE2), thereby interfering with follicular development and oocyte maturation (87). The study found significant abnormalities in lipid metabolism and fatty acid biosynthesis in granulosa cells of patients with PCOS. These metabolic disorders may indirectly interfere with oocyte maturation by affecting the function of granulosa cells. Fatty acid degradation is an important source of ATP during oocyte meiosis, and down-regulation of fatty acid biosynthesize-related genes (such as FASN, SCD, FADS2) in PCOS granulosa cells may lead to insufficient energy supply to oocytes. Thus affecting its maturation (167). Obese women often have adverse outcomes in all aspects of the reproductive system, including preterm birth, congenital abnormalities and neonatal conditions (166). Obesity also leads to the loss of maternal factor Stella in oocytes, which contributes to these reproductive disorders (168). Other studies have found that obesity leads to elevated concentrations of free fatty acids in follicular fluid, causing lipid toxicity that affects oocyte and embryo development (107). Even obese pregnant women can harm fetal development and interfere with the progression of meiotic division in fetal oocytes, with a delay in premeiotic I potentially leading to a reduction in the number of fetal oocytes on a high-fat diet (169). Excessive fat tissue can also cause hormonal imbalances that disrupt the hypothalamic-pituitary-gonadal axis, leading to irregular gonadotropin secretion, which may result in anovulation and irregular menstruation (170). In addition, abnormal lipid metabolism can also lead to disorders of glucose metabolism. Studies have found that the accumulation of visceral fat can damage liver function, hinder the efficient conversion of blood sugar to liver glycogen, thereby exacerbating insulin resistance and interfering with glucose metabolism (171).

Abnormal amino acid metabolism can also affect oocytes. For example: Women with PCOS have severe amino acid metabolism dysfunction. The latest research suggests that elevated levels of branched-chain amino acids (BCAA) in the plasma of women with PCOS and functional defects in the key BCAA metabolic gene PPM1K may disrupt the energy metabolic homeostasis of the follicular microenvironment, thereby inducing phenotypes similar to PCOS—insulin resistance and metabolic syndrome (172, 173). In addition, follicle-stimulating hormone (FSH) regulates glutamine (Gln) levels in the follicular fluid. This regulatory process affects granulosa cell apoptosis through the ASK1-JNK signaling pathway, thereby influencing ovulation in patients with PCOS (145).

The metabolism of the three aforementioned types of nutrients does not exist independently. Among them, lipid metabolism is closely linked to glucose metabolism. Specifically, abnormal lipid metabolism can exacerbate insulin resistance by impairing liver function and hindering the conversion of blood glucose to hepatic glycogen, thereby interfering with glucose metabolism and forming a vicious cycle of metabolic disorder, jointly affecting oocyte maturation and quality.

At present, reproduction-related diseases caused by metabolic abnormalities are becoming increasingly common. Therefore, ART is becoming a more convenient means for infertile patients to achieve pregnancy. However, traditional ART requires ovarian stimulation to increase the number of oocytes, which may lead to ovarian hyperstimulation syndrome and intensify the health and economic pressure (174) on patients. Patients with PCOS, POI and obesity have difficulty generating mature oocytes during direct *in vitro* fertilization, which leads to a lower success rate of ART (175–179). Therefore, this method is not recommended for infertile patients with delayed follicular development. The latest assisted reproductive technology—*in vitro* maturation (IVM)—offers a viable strategy for female fertility conservation by promoting the maturation of a small number of retrieved immature oocytes, which leads to fertilization, embryo transfer, and clinical pregnancy (180). However, IVM technology has a key limitation: it does not ensure that oocytes complete full nuclear maturation and cytoplasmic maturation as they do *in vivo*. Because *in vitro* maturation (IVM) not only lacks the dynamic and physiological hormonal stimulation present *in vivo* (such as FSH, LH, and estrogen gradients), but also lacks precise metabolite concentrations and bidirectional metabolic signaling between cumulus cells and oocytes, and furthermore lacks the three-dimensional follicular structure and paracrine regulation found *in vivo*. As a result, IVM-matured oocytes are generally inferior in quality and developmental potential to naturally matured oocytes *in vivo* (181). The gap was found to be associated with abnormal energy metabolism. IVM oocytes showed weakened fatty acid β-oxidation, overactive glycolysis, and a significantly decreased ATP/ADP ratio, which may be the intrinsic cause of their limited developmental potential (182–184).

In summary, metabolic disorders form the core pathological basis of reproductive disorders by interfering with the normal physiology of oocytes and their microenvironment in multiple dimensions and levels. This reveals the importance of improving overall and local ovarian metabolic health for enhancing natural fertility and the success rate of assisted reproduction. Although new technologies such as IVM offer hope for specific patients, their current effectiveness remains limited by the inability to fully replicate the mature metabolic support *in vivo*, potentially due to the lack of appropriate hormonal signals and critical metabolite levels. In the future, optimizing the oocyte maturation environment by specifically correcting metabolic imbalances will be a key direction for improving the efficiency of fertility treatments.

## 6 Research prospects

For reproductive diseases, there are currently studies on related treatment approaches. Targeted metabolic interventions, such as inositol supplementation or GLUTs regulation, may improve egg quality in women with diabetes or older age (73). Other studies have found that targeting metabolic pathways (such as supplementing GGOH or spermidine) could be a potential therapy for improving fertility in older women (87). In addition, metabolites in the follicular fluid, such as carnitine and glutathione, could serve as biomarkers of oocyte quality and ovarian reserve function as personalized treatments in assisted reproductive medicine (87). Based on the recognition of metabolic defects, metabolic-targeted auxiliary intervention strategies are still being explored. For example, supplementation of NAD+ precursors (such as nicotinamide mononucleotide NMN), carnitine (promoting FAO), coenzyme Q10 (improving mitochondrial function), etc. has shown potential for improving oocyte quality and ovarian function in animal models and some clinical studies (108, 185, 186). These strategies offer new ideas for fertility protection (such as functional optimization of ovarian tissue cryopreservation and resuscitation before chemoradiotherapy) and assisted reproductive technologies (such as optimization of *in vitro* maturation media) (187).

In addition, in the future, multi-omics network models integrating metabolomics, transcriptomics, proteomics and epigenomics will be needed to systematically analyze the metabolic dialogue between oocytes and their microenvironment, as well as how metabolic reprogramming responds to external signals (such as hormones, nutrition) and determines cell fate. The integration of antioxidants during *in vitro* maturation has been shown to improve mitochondrial function and reduce oxidative stress (188), and multiple literature have emphasized that mitochondrial replacement therapy (MRT) can restore the oocyte environment and enhance developmental potential by replacing dysfunctional mitochondria with healthy mitochondria from donor cells, thereby improving reproductive outcomes (189, 190). Therefore, mitochondrial transplantation represents a promising frontier in reproductive medicine, with the potential to improve oocyte quality and prevent the spread of pathogenic mitochondrial DNA mutations (191, 192). Multiple studies have shown that glycine plays a positive rolein promoting oocyte *in vitro* maturation (IVM) and *in vitro* fertilization (IVF) (193, 194). How specific metabolites precisely regulate epigenetic reprogramming (such as DNA methylation and histone modification) during oocyte maturation and early embryonic development remains to be further studied, which is key to understanding the effects of metabolism on intergenerational inheritance. These advancements will contribute to better assessment and improvement of gametes and embryo quality, especially in patients with conditions such as diabetes, insulin resistance, PCOS, POI, and obesity, ultimately increasing the success rate of pregnancy in ART. This progress will not only help families affected by infertility realize their dream of becoming parents, but also support high-quality and healthy growth of the global population.

## 7 Conclusions

Metabolic reprogramming plays a central regulatory role in oocyte growth and development, ensuring the fine requirements of oocytes in terms of energy supply, biosynthesis, REDOX balance, and epigenetic modification through the synergy and conversion of the three metabolic pathways of glucose, lipids, and amino acids. Metabolic disorders such as hyperglycemia, insulin resistance, lipid accumulation and amino acid metabolic dysregulation can significantly disrupt the ovarian microenvironment, impair granulosa cell function, reduce oocyte developmental potential, and are closely associated with reproductive diseases such as polycystic ovary syndrome, early-onset ovarian insufficiency and obesity. Future research should focus on constructing multi-omics metabolic networks of oocytes and microenvironments, elucidating the interaction mechanism between metabolic reprogramming and epigenetic regulation, and exploring strategies such as targeted metabolic interventions, mitochondrial function optimization, and *in vitro* maturation system metabolic support to improve natural fertility and assisted reproductive success rates, ultimately providing new ideas for reproductive health and disease treatment.

## Data Availability

The raw data supporting the conclusions of this article will be made available by the authors, without undue reservation.
